# Nestedness of Ectoparasite-Vertebrate Host Networks

**DOI:** 10.1371/journal.pone.0007873

**Published:** 2009-11-18

**Authors:** Sean P. Graham, Hassan K. Hassan, Nathan D. Burkett-Cadena, Craig Guyer, Thomas R. Unnasch

**Affiliations:** 1 Department of Biological Sciences, Auburn University, Auburn, Alabama, United States of America; 2 Global Health Infectious Disease Research Program, Department of Global Health, University of South Florida, Tampa, Florida, United States of America; 3 Department of Entomology and Plant Pathology, Auburn University, Auburn, Alabama, United States of America; Umea University, Sweden

## Abstract

Determining the structure of ectoparasite-host networks will enable disease ecologists to better understand and predict the spread of vector-borne diseases. If these networks have consistent properties, then studying the structure of well-understood networks could lead to extrapolation of these properties to others, including those that support emerging pathogens. Borrowing a quantitative measure of network structure from studies of mutualistic relationships between plants and their pollinators, we analyzed 29 ectoparasite-vertebrate host networks—including three derived from molecular bloodmeal analysis of mosquito feeding patterns—using measures of nestedness to identify non-random interactions among species. We found significant nestedness in ectoparasite-vertebrate host lists for habitats ranging from tropical rainforests to polar environments. These networks showed non-random patterns of nesting, and did not differ significantly from published estimates of nestedness from mutualistic networks. Mutualistic and antagonistic networks appear to be organized similarly, with generalized ectoparasites interacting with hosts that attract many ectoparasites and more specialized ectoparasites usually interacting with these same “generalized” hosts. This finding has implications for understanding the network dynamics of vector-born pathogens. We suggest that nestedness (rather than random ectoparasite-host associations) can allow rapid transfer of pathogens throughout a network, and expand upon such concepts as the dilution effect, bridge vectors, and host switching in the context of nested ectoparasite-vertebrate host networks.

## Introduction

Increased focus on pathogens that emerge from their usual host populations and affect novel hosts (e.g., humans) is warranted. Several strategies have been used to characterize these pathogens, many of which stem from epidemiological paradigms [1], [2]. Many studies focus on a single species acknowledged to play a key role in a pathogen's transmission. Rarely do such studies consider vector-borne pathogen transmission cycles as properties of whole parasite-host networks [3], [4]. The growing field of disease ecology has begun to resolve this discrepancy by merging the full repertoire of field and experimental research with epidemiological models [5]. Numerous studies have demonstrated the utility of this approach to understand disease dynamics [6]–[8], and these studies have become increasingly important due to the alarming increase in emerging pathogens in recent decades [9]–[12].

Ecologists have developed several measures to characterize ecological network dynamics, including the concepts of modularity [13], connectance [14], degree-strength asymmetry [15], quantitative link density [16], and nestedness [17]–[19]. Nestedness is a factor used to measure the extent to which specialized species (i.e., those with few interactions with other species) interact with increasingly large subsets of generalists (i.e., those with many interactions). Analyses of nestedness are practical tools that require presence/absence data for species interactions at a particular place. Nestedness has been used to examine communities among habitat fragments [20], as well as to characterize network structure of interacting organisms within communities of mutualists [17]–[19], commensals [21], [22], and parasites [23], [24].

At the scale of an infracommunity (within individual hosts) or component community (among populations of a single host species) [25], host-parasite relationships have often been described as being dominated by random associations [26]–[28]. Recent studies have documented nested patterns of interactions [29]–[32]. However, community-level analyses of entire ectoparasite-vertebrate host networks are rare. Although a recent study analyzed the distribution of specialization in host-parasite networks for selected taxa (fleas and rodents, fish and monogeneans) across a single geographic region (north temperate zone) [33], no study has examined entire ectoparasite-vertebrate host networks across a variety of sites.

It has been suggested that interactions based on mutual benefits trend toward nestedness, while those resulting from antagonistic relationships will lead to specialization and compartmentalization [34]. While most large ecological interaction networks have compartments [13], [23], [34], there is disagreement whether parasite-host networks are organized and develop in such a way that might lead to a nested pattern, and a comparative study between these networks at the scale of whole communities (e.g., as in mutualistic networks) [17] is needed [35]. Strong patterns of nestedness in other network interaction types occur due to the presence of generalized species which interact with specialists as well as other generalists. Extreme generalist ectoparasites are known, as well as very specialized species [36], [37]. Thus, we test the hypothesis that generalized and specialized ectoparasites and their vertebrate hosts interact in a way consistent with the pattern of nestedness observed in other network types. Finally, we suggest that nestedness vs. randomness in ectoparasite-vertebrate host networks has the capacity to facilitate the spread of pathogens across taxa.

## Results

Most of our networks (17 of 27) were more nested than the null model simulations using NODF (Table 1). There was no interaction between geographic area or species richness of a network and NODF nestedness (area: R^2^ = 0.11; p>0.05; richness: R^2^ = 0.14; p>0.05). A positive relationship was documented between nestedness (*N*) and species richness of ectoparasite-vertebrate host networks (R^2^ = 0.417; F = 18.777; p<0.0005). Collectively, these data suggest that the completeness of the matrix (in terms of number of species included in the analysis), rather than the size of the area studied, was an important factor influencing nestedness.

**Table 1:** Ectoparasite-vertebrate host networks analyzed for this study.

*Network Location*	*Nestedness* (N)	*Nestedness* (NODF)	*p = *	*Total # species*	*Latitude*	*Host type(s)*	*Ectoparasite type(s)*	*Ref.*
Auburn, AL, USA	0.8494	21.08	0.01	49	Temperate	M†, B, A, R	Mosquitoes	This study
Tuskegee N.F., AL, USA	0.9353	12.58	<0.000	93	Temperate	M†, B, A, R	Mosquitoes	This study
Mississippi, USA	0.8909	41.83	ns	32	Temperate	M,B, A, R	Mosquitoes	This study
St. Catherine's Island, GA, USA	0.9314	17.8	<0.000	92	Temperate	M†, B, R	Ticks, lice, mites, fleas	[68]
Sapelo Island, GA, USA	0.7612	15.15	ns	30	Temperate	M, B, R	Ticks, lice, mites, fleas	[68]
Jekyll Island, GA, USA	0.7902	13.24	ns	36	Temperate	M, B, R	Ticks, lice, mites, fleas	[68]
Cumberland Island, GA, USA	0.8987	20.75	0.01	100	Temperate	M, B, R	Ticks, lice, mites, fleas	[68]
Indiana, USA	0.8913	41.83	<0.000	259	Temperate	M†	Ticks, lice, chiggers, mites, fleas	[60]
Alaska, USA	0.9332	10.06	<0.000	110	Polar	M, B	Ticks, lice, mites, fleas	[69], [70], [71], [72]
New Zealand	0.9636	6.07	<0.000	238	Temperate	M†, B, R	Ticks, lice, mites, fleas mosquitoes, flies	[73], [74]
New Mexico, USA	0.9163	9.35	<0.000	217	Temperate (arid)	M†	Fleas, lice	[75]
Panama	0.9812	2.13	<0.000	645	Tropical	M†, B, R	Ticks, lice, chiggers, mites, fleas, bat bugs, bat flies	[59]
Atlantic Forest, Brazil	0.9763	6.2	<0.000	177	Tropical	M, B	Ticks, lice, mites, fleas, bat bugs, bat flies	[76], [77], [78], [79], [80], [81]
Mountain Zebra N.P., South Africa	0.7864	7.19	<0.000	39	Mediterr-anean	M	Ticks	[82]
Sweden	0.8314	9.68	ns	43	Polar	M†, B	Black flies	[83]
Slovakia	0.883	37.29	<0.000	51	Temperate	M	Fleas	[84]
Paraguay	0.769	10.7	ns	51	Tropical	M	Bat flies	[85]
Britain	0.9049	5.63	ns	137	Temperate	M	Ticks, fleas, chiggers, lice, mites	[86], [87]
Uganda	0.9552	7.97	<0.000	256	Tropical	M†, B, R	Ticks, fleas, lice, tsetse flies	[88], [89], [90], [91]
Gannet Islands, Labarador	0.4864	57.49	ns	21	Polar	B	Ticks, lice	[92]
New South Wales	0.6281	38.93	ns	59	Temperate (marine)	F	Monogeneans, isopods,copep-ods	[93]
Pacific Canada	0.6314	11.79	ns	58	Temperate (marine)	F	Monogeneans, isopods,copep-ods	[93]
South Carolina, USA	0.8555	6.07	ns	81	Temperate	M, B, R	Ticks, fleas, mites, chiggers	[94]
Utah, USA	0.9519	7.88	<0.000	244	Temperate (arid)	M, B, R	Ticks, chiggers, fleas, mites	[95], [96], [97], [98], [99]
Queensland, Australia	0.977	4.81	<0.000	389	Trropical	M†, B, R	Ticks, fleas, chiggers, batflies	[100], [101], [102], [103]
Tasmania, Australia	0.9197	9.84	<0.000	164	Temperate	M†, B, R	Ticks, fleas, chiggers, batflies	[100], [101], [102], [103]
Madagascar	0.9023	6.52	0.02	88	Tropical	M†	Ticks	[104]

M = mammals, B = birds, R = reptiles, A = amphibians, and F = fish.

† Indicates that network specifically included humans.

**Figure 1:**
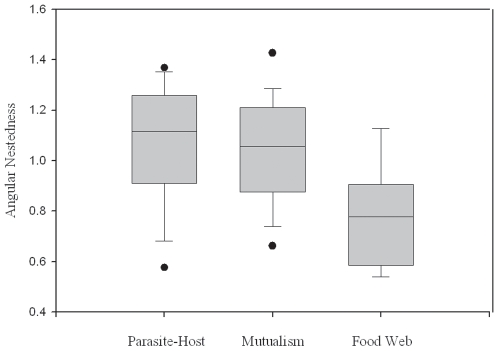
Box-plots of mean nestedness (*N*). Comparison between previously published calculations of plant-pollinator/seed dispersal (mutualism) networks, food webs [17], and ectoparasite-vertebrate host networks (this study).

**Figure 2:**
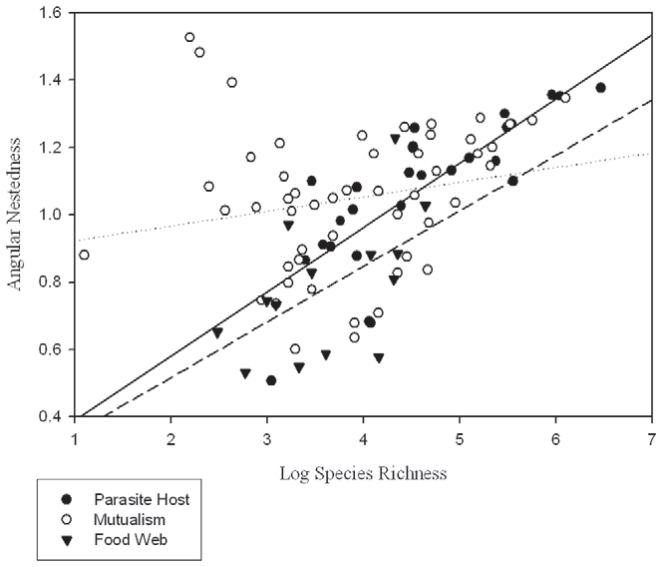
Regressions of nestedness (*N*; angular transformed) on species richness (log transformed). Closed circles  =  ectoparasite-vertebrate host networks (this study); open circles  =  mutualisms (plant-pollinator and plant-seed disperser networks) [17]; closed triangles  =  food web networks [17].

In our comparison to previous work using the nestedness estimate *N*, We found a significant main effect of network type on degree of nestedness (F_2,13_ = 6.20; p = 0.003) such that nestedness of ectoparasite-vertebrate host networks did not differ significantly from mutualistic plant-pollinator or seed dispersal networks, but did differ from food webs (Fig. 1). However, this analysis was confounded by a significant interaction between species richness and network type (F_2,13_ = 9.26; p<0.0001). There were significant positive correlations between species richness and degree of nestedness for the networks based on antagonistic relationships (ectoparasite-vertebrate host: R^2^ = 0.61; F = 39.3; p<0.0001; food web: R^2^ = 0.305; F = 5.273; p = 0.04), but no correlation for networks based on positive relationships (mutualism: R^2^ = 0.043; F = 2.216; p>0.05; Fig. 2). Comparison to plant-pollinator networks available through the Interaction Web Database also yielded no significant differences using the nestedness indicator NODF (F = 0.768; p = 0.469).

**Figure 3:**
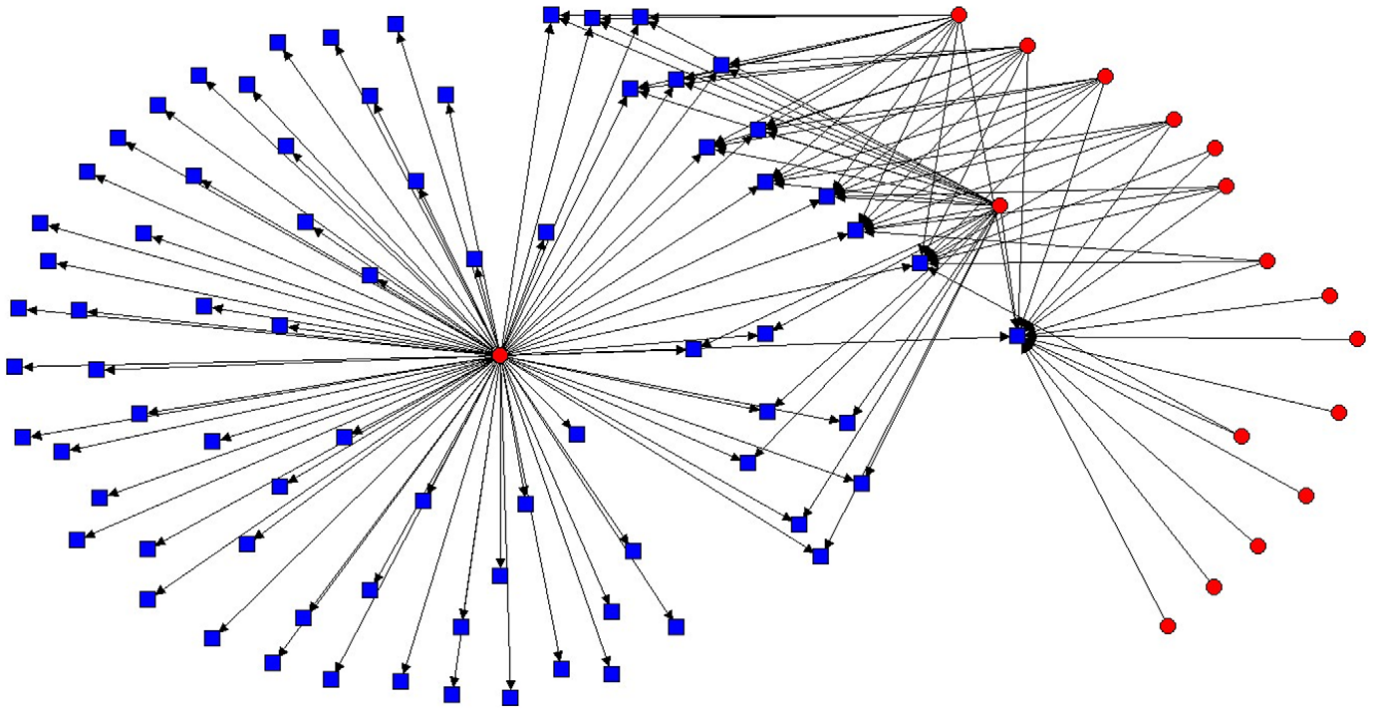
Hypothetical network of perfect nestedness for 17 ectoparasites and 76 hosts. Drawn using UCINET.

**Figure 4:**
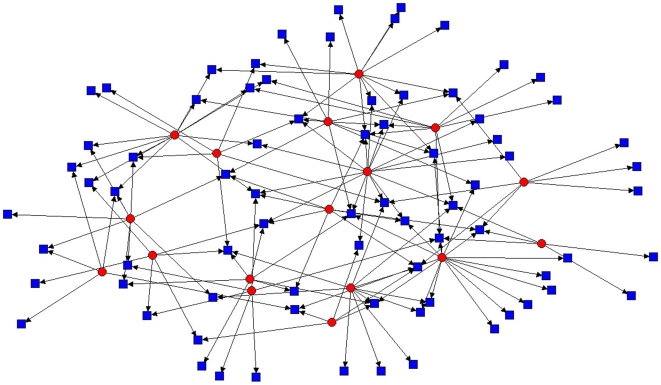
Hypothetical network of random interactions for 17 ectoparasites and 76 hosts. Drawn using UCINET.

**Figure 5:**
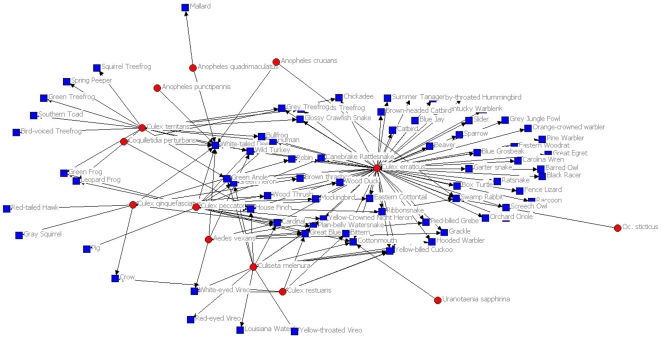
Observed network of mosquitoes and their vertebrate hosts. Network from Tuskegee National Forest, Alabama, USA, representing 17 actual mosquito species and their 76 vertebrate hosts. Red dots represent mosquitoes and blue squares represent vertebrate hosts. Drawn using UCINET.

Within the hypothetical, perfectly nested TNF network, only a single mosquito was needed to retain connections to all hosts (Fig. 3). Randomized associations of this same matrix reduced dominance of any one host or parasite and, in the example presented here (Fig. 4), required 15 parasites to retain connections to all hosts. The observed diagram for this network (Fig. 5) was more similar to the extreme of complete nestedness than it was to the extreme of random expectation. This resulted from a dominant mosquito (*Culex erraticus*) that visited most hosts, and a dominant host (White-tailed deer) that was visited by more mosquitoes than any other host. Because of significant nesting, only six of the 17 mosquitoes in this network were required to maintain connections to all known hosts.

## Discussion

We detected significant nestedness in most ectoparasite-vertebrate host networks. This indicates that specialized ectoparasites usually interact with hosts that attract many ectoparasites (‘generalist’ hosts), and generalist ectoparasites interact with these hosts as well as those that attract fewer ectoparasites (‘specialist’ hosts). Networks analyzed were as species rich (21–645 species), and were as geographically diverse (including networks from five continents) as previous attempts to characterize nestedness in other types of species interactions [17]–[19]. Despite the diversity of latitudinal, ecosystem, and taxonomic affiliations of these networks, considerable nestedness appears to be a consistent rule in ectoparasite-vertebrate host networks.

Moreover, our results were robust with respect to passive sampling and other problems associated with nestedness estimates, which can inflate the results of nestedness calculations [38], [39]. Sampling artifacts could still be responsible for a portion of the trends observed because parasite species richness and host abundance covary in a way that is difficult to tease apart [40]. However, biologically meaningful patterns occur between hosts and parasites despite this dilemma [33], [41], and nestedness analyses are robust to variation in sampling effort [42]. Our mosquito-host networks were also statistically nested despite the fact that mosquitoes were the collectors of host samples. This offers an interesting qualitative comparison between host versus ectoparasite sampling and their potential biases; in both situations, nestedness detected was significantly higher than null model simulations.

These results underscore the remarkably similar properties that have been attributed to networks with interacting components, from human social systems to mutualistic networks of plants and pollinators [43]. These networks are often characterized by asymmetric connections between network members, with generalist species interacting with each other, and specialized species (e.g., those with few interactions) usually interacting with generalists [34], [35]. A study of flea-rodent networks from north temperate zones revealed these patterns hold for ectoparasites [33], and our data corroborate and expand upon this. Mutualistic and parasitic networks are on opposite ends of the interaction spectrum in terms of the relationship of the participants (from +/+ to +/−), and nonetheless they appear to share a similar network structure. Although counterintuitive, the overall similarity and low interaction intimacy of plant/host visitation by pollinators/ectoparasites—and their subsequent co-evolutionary interactions—probably lead to this structure [44].

Various factors suggested to play a role in the development of nested mutualistic networks [45], [46] probably also contribute to the nested structure documented here. Much of the nestedness detected in our networks is probably due to abundance and sampling interactions [33]; the most abundant species typically are responsible for large cores of interactions, and rarer, more specialized species are more likely to “sample” the most abundant hosts. Phenotypic trait matching between ectoparasites and host defenses could contribute to nested patterns. The mouthparts, attachment organs, behavior, and morphology of ectoparasites exhibit generalized and more specialized patterns for obtaining blood from a variety of hosts or specific hosts [47]. Hosts have also developed variable defenses which could lead to nested preferences among ectoparasites [48], [49]. Spatiotemporal distribution of ectoparasites and their hosts (e.g., some ectoparasites and potential hosts are not coincident in space or time) and the existence of ‘forbidden interactions’ (mosquitoes do not feed upon fish or salamanders at our study site) [50] may possibly play a role as well. Finally, there is evidence of phylogenetic concordance in ectoparasite-host interactions [51], and future studies which incorporate phylogenetic information will likely determine that this is an essential factor in the nesetedness of these networks [52].

Our comparison using a network analysis which takes into account varying network properties (NODF) showed no differences between ectoparasite-vertebrate host networks and plant-pollinator networks. This analysis and others have determined nestedness in parasite-host networks [23], [24], suggesting that a dichotomy between network structure may occur between different interaction intimacies [44], rather than differences in the interaction types (i.e., +/+ vs. +/−). It is therefore likely that nonsymbiotic mutualistic and antagonistic interaction networks are simultaneously compartmentalized and nested [13], [17], [23], [44]. Our results confirm the nestedness of antagonistic nonsymbiotic networks, although the networks we analyzed represent a range of interaction intimacies ranging from low (mosquitoes) to relatively high (ticks, lice). Qualitatively, we suggest that these networks are compartmentalized, since most ectoparasite groups appear to form their own nested subcompartments. Future research should seek to determine if ectoparasite-vertebrate host networks are made up of large nested networks composed of nested subcompartments linked by generalists, and focus should also be directed toward endoparasite-host networks to confirm that symbiotic vs. nonsymbiotic networks exhibit different properties.

Our results have implications for understanding disease transmission cycles. In the specific case of mosquitoes at Tuskegee National Forest (TNF), nesting assures that novel diseases, such as West Nile virus, have the potential to spread widely among vertebrate hosts. This prediction is associated with the fact that nesting results from a core ectoparasite (*Culex erraticus*) that feeds on the majority of vertebrate hosts and a core host (White-tailed deer) that is fed upon by the majority of mosquitoes. These core taxa greatly reduce the number of linkages needed to unite all taxa within the network. Thus, only six mosquitoes are required to retain all interactions of the TNF network whereas 15 mosquitoes are required for a randomized version of the same network. The concept of compartmentalized nestedness predicts that pathogens likely coevolve with hosts and ectoparasites within specific compartments, but the possibility for escape into additional or all other compartments is possible due to the high connectance of the network. Zoonoses (diseases caused by pathogens which normally cycle through wildlife) can thus be the result of compartment-spanning or compartment-shifting pathogens [53], [54], and network analysis may lead to discovery of likely conditions that exacerbate such phenomena (e.g., during peaks of host or ectoparasite abundance).

Increased nestedness in larger ectoparasite-vertebrate host networks implies that species richness actually leads to increased connectance between individual species in the network, greatly facilitating the spread of disease unless member hosts, especially core hosts, have reduced capacity to amplify and allow transmission of a pathogen (e.g., decreased reservoir competence). Thus, the transmission pattern of vector-born pathogens is likely determined by high connectivity between hosts and ectoparasites due to nestedness, counterbalanced by the existence of nested compartments of resistant hosts that are dead ends for pathogens (expanding the “dilution effect” hypothesis) [4].

Finally, disease ecologists often attempt to understand and mitigate vector-born pathogens from the host point of view, and network analyses are a new way of looking at this network interaction instead as a double-edged sword. For example, vector-born diseases are often thought of as the result of ectoparasites that have broad host preferences (so-called bridge vectors), which occasionally spread diseases to humans [55]–[57]. Network analyses reveal the whole story: bridge vectors are usually abundant and catholic in their host preference, however, attention from these ectoparasites comes as the inevitable consequence of being a large and/or abundant potential host. Current theory regarding host switching by ectoparasites as a driver for disease emergence [53], [57] should consider this; host switching may have less to do with ectoparasite host switching and more to do with host population dynamics (e.g., surges in host abundance and/or availability may lead to recruitment of new ectoparasites). Fortunately, since ectoparasite-vertebrate host networks have consistent network properties, findings from well-studied model systems (e.g., lyme disease, ticks, and their hosts) [58] should have reliable crossover to less well-studied networks which harbor vector-born diseases. Thus, nestedness analyses should be seen as a new step toward understanding the complexity of these networks in such a way that new threats—such as emerging infectious diseases—can more quickly be characterized, understood, and effectively managed.

## Methods

### Ectoparasite-Vertebrate Host Networks

Twenty-six ectoparasite-vertebrate host matrices were assembled from lists of interacting species available from the literature (Table 1). Each list was generated for a specified contiguous region. Methodology for these studies typically included capturing vertebrates and identifying ectoparasites extracted from them [59], [60]. These studies described regions that ranged in size from entire countries (e.g. Panama, South Africa) to smaller regions within countries (e.g. Gannet Islands, Labrador). Therefore, these matrices were composed of multiple component communities [25]. This approach is analogous to the methodology of previous nestedness analyses of plants and their pollinators or seed dispersers [17].

Ectoparasites and their vertebrate hosts were included within interaction matrices only if species-level identifications were available. This approach probably underestimates total species richness within each network, since small ectoparasites frequently are identified only to genus. This procedure might underestimate nestedness since many specialist ectoparasites probably remain undescribed.

To these literature records we added three mosquito-host networks derived from our studies of vectors of West Nile virus (WNV) and eastern equine encephalitis virus (EEEV) in Mississippi and Alabama, USA. In these studies we developed extensive host lists for diverse mosquitoes using molecular analysis of mosquito bloodmeals. In brief, we collected blood-engorged female mosquitoes from field sites and identified species of vertebrate hosts of individual mosquitoes using Polymerase Chain Reaction (PCR) amplification of host DNA in blood meals. Resulting sequences were matched to sequences in GENBANK via BLAST to determine the likely host species (98% sequence match). Additional details of the procedures used to identify host bloodmeals can be found elsewhere [50], [61], [62].

### Measure of Nestedness

Nestedness was determined from each network using ANINHADO 3.0 software [63]. This program maximally packs each matrix representing a network, and then calculates an isocline of perfect nestedness for the given size and shape of a matrix. Within this matrix the distance of each interaction (fill) from the isocline is calculated, averaged, and used to create an index (*T*) that varies from 0 (perfectly nested) and 100. Following 17, we then calculated *N* = (100–*T*)/100 so that increasing values of the index represented increasing nestedness and to facilitate comparison to analyses of other published networks.

There has been a recent surge of interest in refining nestedness analyses such that they better reflect the original concept of nestedness [63]. Therefore, we also determined a new measure of nestedness (e.g., NODF) using ANINHADO 3.0 software [63]. This measure accounts for certain flaws in other nestedness estimates that have inappropriate interactions with matrix properties such as size and shape. NODF considers matrix row and column fill differences, as well as the overlap of row and column presences [63]. Although this metric is clearly superior to absolute nestedness temperature estimates, we provide analyses using *N* to allow comparison to previous work. However, due to statistical problems associated with *N* [63], we considered analyses involving NODF the test of our hypothesis, and only these analyses are considered as such in our results and discussion. We compared *N* of our networks to published estimates of nestedness for mutualisms and food webs from 17. NODF values were compared to 32 plant-pollinator networks from around the world available from the Interaction Web Database [64], [65].

### Data Analysis

Calculations of nestedness were tested against a null model generated by Monte-Carlo simulations (1000 permutations) within program ANINHADO [63]. Nestedness might result from passive sampling rather than assemblage structuring [39], especially for ectoparasite-vertebrate host networks in which common ectoparasites and hosts might be sampled more frequently than rare ones. To account for this, we chose a null model which considers passive sampling [39]. This null model simulation randomly fills cells in proportion to the row and column totals of each interacting species.

Two extreme outliers (nestedness values were > two standard deviations above the mean value) [66] were removed from the dataset leaving a sample of 27 networks. These were ectoparasite-vertebrate host networks with highest and lowest species richness. Nestedness values were angular transformed and the corresponding value of species richness of each network was log transformed to achieve normality [18]. Potential confounding effects of geographic area and species richness on nestedness were analyzed using regression. Ectoparasite-vertebrate host networks were compared to other ecological networks [17] using an ANCOVA with network type (host-parasite, food web, or plant-pollinator/disperser) as the main effect, nestedness as the dependent variable, and species richness as the covariate. NODF values for ectoparasite-vertebrate host networks were similarly compared to plant-pollinator networks available from the Interaction Web Database.

To visualize potential patterns of interactions at the Tuskegee National Forest site, we used UCINET software to create ball-and-stick models for a fully nested, a random, and the observed set of interacting species. We then calculated the minimum number of mosquitoes required to sample blood from the vertebrate hosts in fully nested, random, and the observed mosquito-host networks [67]. In all analyses, α was set at 0.05.
